# A therapeutic approach to pantothenate kinase associated neurodegeneration: a pilot study

**DOI:** 10.1186/s13023-024-03453-x

**Published:** 2024-11-28

**Authors:** Alessandra Pereira, Carolina Fischinger Moura de Souza, Mónica Álvarez-Córdoba, Diana Reche-López, José Antonio Sánchez-Alcázar

**Affiliations:** 1 Pediatrics Service, Hospital Moinhos de Vento, Porto Alegre, RS Brazil; 2 Medical Genetics Service, Hospital de Clínicas de Porto Alegre, Casa Dos Raros, Porto Alegre, RS Brazil; 3 Andalusian Centre for Developmental Biology-CSIC-Pablo de Olavide University, 41013 Seville, Spain

## Abstract

**Background:**

Neurodegeneration with brain iron accumulation (NBIA) is a group of genetic neurological disorders frequently associated with iron accumulation in the basal nuclei of the brain characterized by progressive spasticity, dystonia, muscle rigidity, neuropsychiatric symptoms, and retinal degeneration or optic nerve atrophy. Pantothenate kinase-associated neurodegeneration (PKAN) is one of the most widespread NBIA disorders. The diagnosis of PKAN is established with clinical features and the “eye of the tiger” sign identified on brain MRI and the identification of biallelic pantothenate kinase 2 (PANK2) pathogenic variants on molecular genetic testing. PANK2 catalyzes the first reaction of coenzyme A (CoA) biosynthesis, thus, altered PANK2 activity is expected to induce CoA deficiency as well as low levels of essential metabolic intermediates such as 4′-phosphopantetheine which is a necessary cofactor for critical proteins involved in cytosolic and mitochondrial pathways such as fatty acid biosynthesis, mitochondrial respiratory complex I assembly and lysine and tetrahydrofolate metabolism, among other metabolic processes.

**Methods:**

In this manuscript, we examined the effect of a multitarget complex supplements (pantothenate, pantethine, omega-3 and vitamin E) on in vitro patient-derived cellular models and the clinical outcome of the adjuvant supplements in combination with the baseline neurological medication in three PKAN patients.

**Results:**

Multitarget complex supplements significantly reduced iron accumulation and increased *PANK2* and ACP expression levels in the cellular models derived from all three PKAN patients. In addition, the adjunct treatment to the standard neurological medication improved or stabilized the clinical symptoms of patients.

**Conclusions:**

Our results suggest that multitarget complex supplements can be clinically useful as augmentation therapy for PKAN patients harboring pathogenic variants with residual enzyme levels.

*Trial registration*: CAAE: 58219522.6.0000.5330. Registered 25 May 2022—Retrospectively registered, https://plataformabrasil.saude.gov.br/visao/pesquisador/gerirPesquisa/gerirPesquisaAgrupador.jsf.

**Supplementary Information:**

The online version contains supplementary material available at 10.1186/s13023-024-03453-x.

## Introduction

Pantothenate-kinase-associated neurodegeneration (PKAN) is a rare disease caused by pathogenic variants in the *PANK2* (*pantothenate kinase 2*) gene. *PANK2* pathogenic variants lead to symptoms as dystonia, rigidity, choreoathetosis, retinal degeneration or optic atrophy, neuropsychiatric abnormalities and early death. Anatomopathological features are often associated with iron accumulation in the globus pallidus and to a lesser degree in substantia nigra and adjacent regions, and the presence of axonal dilations (spheroids bodies) in the central nervous system, corresponding to damaged neurons [1]. The characteristic imaging hallmark of PKAN is the “eye-of-the tiger” sign connected to focal iron accumulation in the globus pallidus on T2-weighted magnetic resonance imaging (MRI), manifesting peripheral hypointensity surrounding central hyperintensity in the globus pallidus [2].

Pathogenic variants in *PANK2* gene, that codes for an essential enzyme in coenzyme A (CoA) biosynthesis, are one of the most common subtype of NBIA; it accounts for almost the 50% of cases [3]. The phenotypic spectrum of PKAN includes classic PKAN and atypical PKAN. Classic PKAN is characterized by starting in early childhood (before 6 years of age in 88% of cases) of progressive dystonia, rigidity, dysarthria, choreoathetosis, and pigmentary retinal degeneration. Atypical PKAN is characterized by later onset (age > 10 years), marked speech defects, psychiatric disturbances, and a slower progression of disease [4, 5].

Of the four members of the pantothenate kinase gene family—*PANK1a*, *PANK1b*, *PANK2*, and *PANK 3*—only *PANK2* is the gene that causes PKAN. The PANK2 enzyme uses ATP to convert (R)-pantothenate into (R)−4'-phosphopantothenate, and it is found in the intermembrane space of mitochondria [6]. The enzyme alteration causes coenzyme A deficiency, mitochondria dysfunction and low energy production, intracellular iron accumulation, alterations in cell membranes renewal and impaired protection against oxidative damage, which provokes lipid peroxidation and pathological changes of cell membranes, and eventually cell demise [7, 8]. Altered mitochondrial membrane potential and defective mitochondrial respiration have been demonstrated in *PANK2*-defective neurons derived from KO mice [9] and in cellular models derived from PKAN patients [10–12]. Nevertheless, there is still much to learn about the specific pathological mechanisms underlying PKAN.

Apart of metabolic alterations including impairment of the citric acid cycle, sterol and steroid biosynthesis, heme biosynthesis, amino acid synthesis, and β-oxidation [13], low CoA levels particularly in mitochondria also affect the 4′-phosphopantetheinylation of essential proteins for mitochondrial function and cell homeostasis [14, 15]. The rationale is that CoA is the supply source for the 4'-phosphopantetheine moiety needed for the posttranslational 4'-phosphopantetheinylation required to activate specific proteins. Thus, multi-enzyme complexes which sequentially catalyse several reactions are often dependent on the covalent binding of a 4’-phosphopantetheine cofactor to specific proteins. This protein carries metabolic intermediates in the process of different enzymatic reactions. In mammals, the transfer of the 4’-phosphopantetheinyl cofactor from coenzyme A to specific proteins takes place following protein biosynthesis as a post-translational modification [16]. Thus, 4’-phosphopantetheinylation is necessary for the transformation enzymes into their full-active forms [16].

In our previous works, using cellular models derived from PKAN patients, we confirmed the hypothesis that CoA deficiency caused by *PANK2* variants affects the expression levels and activity of key mitochondrial proteins harboring a 4′-phosphopantetheiny cofactor such as mtACP (mitochondrial acyl carrier protein), ALDH1L2 (mitochondrial 10-Formyltetrahydrofolate Dehydrogenase) or AASS (alpha-aminoadipic semialdehyde synthase) [14]. Reduced mtACP levels also affect the lipoylation of 2-oxoaxid dehydrogenases such as PDH-E2 (pyruvate dehydrogenase-E2).

Despite extensive research efforts in the development of new treatments, there is still no successful treatment to stop the progression of neurodegeneration in PKAN. Thus, alternative therapeutic research strategies are needed. Although great efforts have been made to model the disease in mice, they have produced incomplete phenotypic alterations such as iron accumulation in the brain and movement disorder, possibly because the location of PANK2 in mitochondria has only been reported in humans and primates [17].

Observations from previous studies in cellular models suggest that pantothenate can increase PANK2 expression levels in patient-derived fibroblasts harboring specific variants [10]. Furthermore, using iNs generated by direct reprogramming of mutant fibroblasts, the positive effect of pantothenate was also confirmed. These observations lead us to propose that cellular models are useful tools for identifying patients with *PANK2* variants with residual enzyme activity that respond in vitro to pantothenate supplementation and raise the possibility of treatment using a high dose of pantothenate or pantothenate derivatives with better bioavailability. Moreover, information on multiple types of *PANK2* gene mutations and their sensitivity to pantothenate supplementation will be necessary to pave the way toward personalised therapies in PKAN. Furthermore, personalised screening strategies can facilitate the detection of more pharmacological chaperones that can stabilise the expression levels and activity of the mutant enzyme. Previous results of our group have identified several compounds which are indeed capable of restoring PANK2 expression levels and improving pathophysiological alterations in fibroblasts derived from PKAN patients [10, 14, 18].

In this manuscript, we report a successful adjuvant treatment with a multitarget complex supplements in combination with the standard neurological medication on symptoms and disease progression in three patients harboring novel variations in the *PANK2* gene. The adjunct treatment was designed for increasing PANK2 expression levels by enhancing pantothenate concentration, the substrate of the mutant enzyme (pantothenate and pantethine) and reducing oxidative stress and lipid peroxidation (Omega 3 and vitamin E).

## Methods

### Ethical approval and consent for publication

A single-arm, open-label study was conducted. All subjects received the multitarget complex supplements during at least 24-week period of treatment. This study was approved by the ethics committee of Hospital Virgen del Rocío and Virgen Macarena (BRAINCURE16) and by the Ethics Committee of Moinhos de Vento Hospital (CAAE Code: 58,219,522.6.0000.5330) and all the parents or legal guardians of subjects signed the written informed consent forms before any procedure was done.

Neurological and geneticist specialists conducted neurological examinations and clinical assessments. Diagnostic criteria for PKAN were set according to standard criteria [19]. Additionally, the patient with PKAN underwent high-field (3.0 T) MRI and laboratory tests.

### Reagents

Monoclonal Anti-α-tubulin antibody, Prussian Blue, sodium pantothenate and trypsin were purchased from Sigma Chemical Co. (St. Louis, MO). Anti-mitochondrial acyl carrier protein (mtACP) antibody was purchased from Invitrogen/Molecular Probes (Eugene, OR). Anti-PANK2 antibody, complex 1 activity kit, and PDH activity were purchased from Abcam (Cambridge, UK).

A cocktail of protease inhibitors (complete cocktail) was purchased from Boehringer Mannheim (Indianapolis, IN). The Immun Star HRP substrate kit was from Bio-Rad Laboratories Inc. (Hercules, CA).

### Cell culture

We used primary skin fibroblasts from two unaffected subjects (controls 1 and 2, one adult and one neonatal) purchased from ATCC (American Type Culture Collection) and three PKAN patients. The fibroblasts were grown in DMEM (Lonza) supplemented with 10% FBS (Lonza), 100 mg/ml streptomycin, 100 U/ml penicillin and 4 mM l-glutamine (Sigma). All the experiments were performed with fibroblasts cell cultures with a passage number < 10.

### Immunoblotting

Western blotting was performed using standard methods described in previous manuscripts of the research group [10]. After protein transfer, membranes were incubated with various primary antibodies diluted 1:1000, and then with the corresponding secondary antibody coupled to horseradish peroxidase at a 1:10,000 dilution. Specific protein complexes were identified using the Immun Star HRP substrate kit (Biorad Laboratories Inc., Hercules, CA, USA). Protein loading was assessed by Ponceau staining and actin expression levels. If the molecular weight of proteins did not interfere, membranes were re-probed with different antibodies. In the case of proteins with different molecular weights, membranes were cut and incubated with specific antibodies.

### *Iron* determination

For the detection of intracellular iron in fibroblasts, 50,000 cells were seeded per well in 6-well plates and cultured for 48 h at 35 °C with 5% CO_2_ to achieve a confluence of approximately 60–70%. After incubation, the cells were washed with PBS and fixed with 4% paraformaldehyde for 15 min at room temperature. Following fixation, the cells were incubated with Prussian Blue solution for 15–30 min, allowing the formation of blue-stained iron complexes. After three washes with distilled water, the samples were evaluated by Prussian Blue staining and quantified by bright-field microscopy.

Quantification analysis was performed by using the ImageJ software. Iron content in cell extracts were also determined by ICP-MS [20]. Iron concentration is expressed as nmol Fe^2+^/μg protein. Data are presented as means ± SD (standard deviation), n = 3 in all cases.

### Lipid peroxidation

Lipid peroxidation was evaluated using 4,4-difluoro-5-(4-phenyl-1,3-butadienyl)−4-bora-3a,4a-diaza-s-indacene-3-undecanoic acid (BODIPY® 581/591 C11), a lipophilic fluorescent dye [21, 22]. Cells were incubated with 1–5 µM BODIPY® 581/591 C11 for 30 min at 37ºC. Control fibroblasts treated with 500 µM Luperox® for 15 min were used as positive control of lipid peroxidation. Lipid peroxidation in fibroblasts was evaluated by an Axio Vert A1 fluorescence microscope with a 20X objective. Images were analysed with Fiji-ImageJ software. Results were expressed as the ratio of the oxidized BODIPY-C11 signal (green) to reduced BODIPY-C11 signal (red).

### PDH activity

PDH complex activity in whole cells was measured using the Pyruvate dehydrogenase (PDH) Enzyme Activity Dipstick Assay Kit (ab109882, ABCAM, Cambridge, MA, USA) according to manufacturer’s instructions. Three biological replicates were used per measurement. Results are expressed as enzyme activity with respect to control. The signal intensity was analyzed by a Molecular Imager ChemiDoc™ MP Imaging System (Bio-Rad, Hercules, CA, USA).

### Real-time quantitative PCR (qPCR)

*PANK2* gene expression in fibroblasts was analysed by qPCR using mRNA extracts. mRNA was isolated with TrizolTM (Invitrogen, Carlsbad, CA, USA), following manufacturer’s instructions. RNA was retrotranscribed using Iscript cDNA synthesis Kit (Bio-Rad,Hercules, CA, United States) to obtain complementary DNA (cDNA). qPCR was performed using TB Green™ Premix Ex Taq™ (Takara Bio Europe S.A.S., Saint-Germain-en-Laye, France). CFX Connect Real-Time PCR Detection System (Bio-Rad, Hercules, CA, USA) was used to detect accurate quantification of gene expression. *PANK2* primers were 5’ TTCCCACTCATGACATGCCT-3’ (Forward primer) and 5’-GTGACCGTCCATTGAATCCG-3’ (Reverse primer) amplifying a sequence of 215 nucleotides. Actin was used as a housekeeping control gene and the primers were 5’- AGAGCTACGAGCTGCCTGAC −3’ (Forward primer) and 3’- AGCACTGTGTTGGCGTACAG −5’ (reverse primer).

### Statistics

We used non-parametric statistics that do not have any distributional assumption, given the low reliability of normality testing for small sample sizes used in this work. To compare parameters between groups, variables were evaluated using Mann–Whitney test for two groups and Kruskal–Wallis test to compare multiple groups. All results are expressed as mean ± SD of 3 independent experiments and a p-value < 0.05 was considered as statistically significant. Statistical analyses were made with GraphPad Prism 7.0 (GraphPad Software, San Diego, CA USA).

## Case reports

The first patient (P1), an 8-year-old girl, with of a healthy, non-consanguineous couple with no family history of genetic condition. The medical history was referred with gait difficulties and frequent falls since the age of 3. Ophthalmologic evaluation showed no visual impairment.

The neurological examination showed hypokinesia, bradykinesia and limb rigidity, which was more pronounced on the right upper limb and dysarthria. Biceps reflex and triceps reflex in both upper limbs were weak. Deep tendon reflexes in both lower limbs were normal. Romberg signs and cerebellar signs were absent. Laboratory tests, including routine blood counts, peripheral blood cell morphology and properties, biochemistry were normal.

**Fig. 1:**
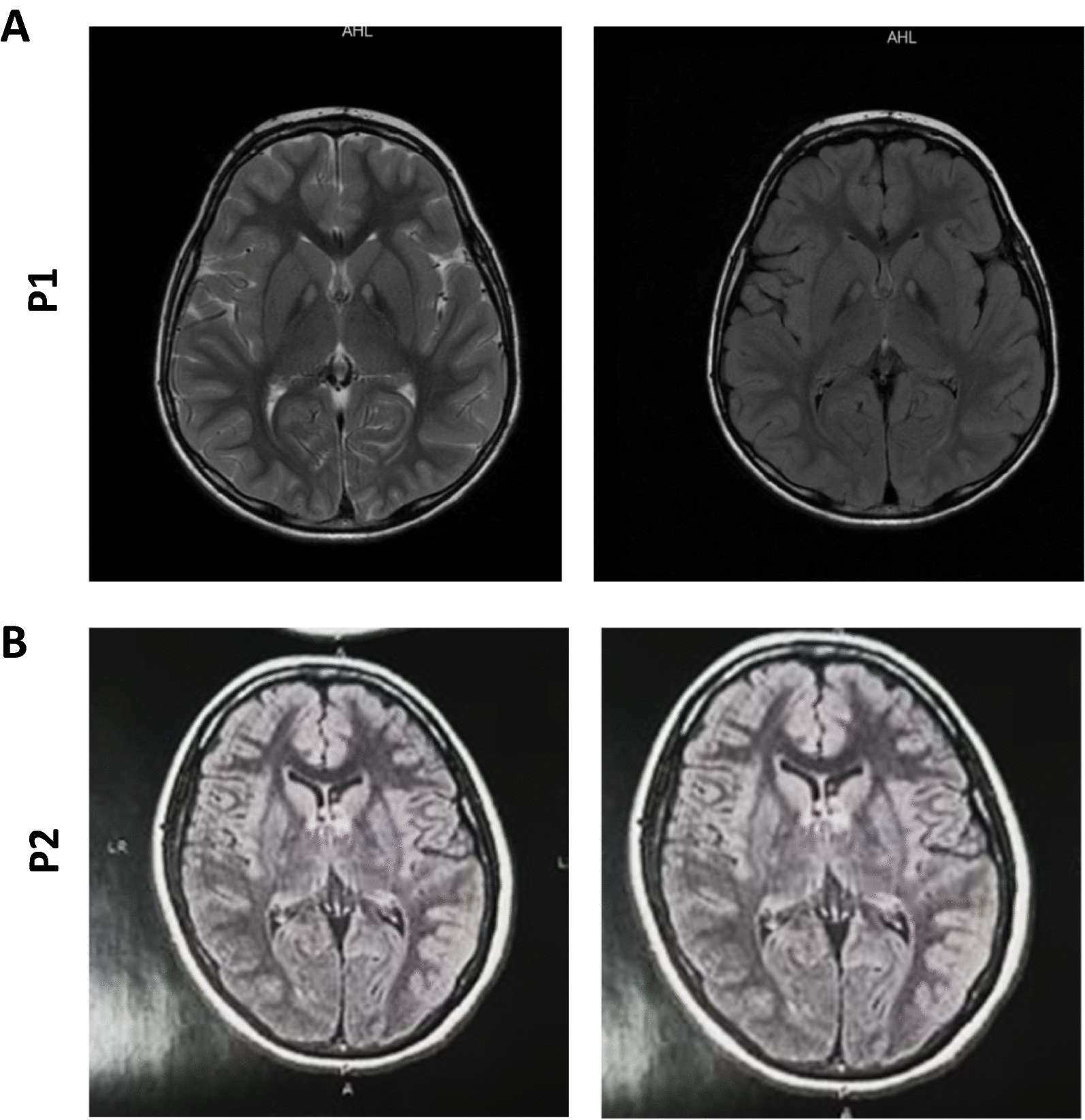
(**A**) P1: MRI (T2 flair and T2 FSE) showing signal hyperintensity due to gliosis in the antero-medial region of both globus pallidus, surrounded by hypointense areas secondary to iron deposits (“eye-of-the-tiger” sign). (**B**) P2: Presence of hypersignal in FLAIR sequence, affecting the globus pallidus bilaterally, delimited by a hyposignal halo, more evident in the sequence for magnetic susceptibility and diffusion, characterizing a "eye-of-the-tiger" appearance

Brain neuroimaging findings showed that T2- weighted and fluid-attenuated inversion recovery images revealed “eye-of-the-tiger” signs, i.e., bilateral symmetrical hypointensity signs in the globus pallidus with central hyperintensity signs (Fig. 1A). Brain susceptibility weighted imaging demonstrated a bilateral symmetrical low signal, indicating brain iron accumulation in the globus pallidus.

Two compound heterozygous variants, based on transcript reference in hg19, were detected: c. [1168-3C > G] and c.[1263dupG]. The first is a base substitution from C to G at position −3 of intron 7 (c. [1168-3C > G]), while the second is a duplication of a guanine at position 1263 in the genomic reference sequence (c.[1263dupG]). The variants mentioned are of the splice site type (c.1168-3C > G) and duplication type (c.1263dupG). The variant c.[1168-3C > G] occurs at a splicing site, affecting messenger RNA processing, while the variant c.1263dupG involves the duplication of a guanine in a specific codon, leading to changes in the gene reading frame (p.Gln422AlaFs*21).

The second patient (P2), a 21- year-old female presented with parkinsonism and progressive generalized dystonia that had started 7 years earlier. Her developmental history and ophthalmological examination were unremarkable with no evidence of visual impairment and retinopathy. The neurological examination showed hypokinesia and limb rigidity. Both muscular and tendinous reflexes appeared diminished. Sole reflexes were normal. Her speech difficulties had gradually become more severe, and it was sometimes difficult to understand. Limb reflexes were brisk, and she was unable to walk without support, and her gait was characterized by end block posture with impaired balance and a tendency to fall backwards, lacking postural reflexes and severe pronation of the right foot. The patient’s cognition appeared normal but revealed symptoms of depression. There was no difficulty with calculation or short-term memory. Her mini-mental state examination score was 30/30.Tongue and soft palate were strong at midline. MRI of P2 showed "eye-of-the-tiger" sign (Fig. 1B). Biochemistry and hematology variables were normal. A genetic study confirmed two pathogenic heterozygous variations in the PANK2 gene c. [950G > C] (p.Gly317Ala); c.[1231G > A] (p.Gly411Arg).

The third patient (P3) was a 40 years-old male, presented with parkinsonism as the initial symptoms besides dystonia in lower limbs since the age of 20. The neurological examination revealed the presence of dysarthria, focal dystonia involving the left hand, and facial tics, as well as the presence of hyperreflexia and spasticity of the lower limbs, with flexor plantar reflex. Tests for strength, coordination and sensitivity were unremarkable. No chorea or myoclonus was evident. The neuro-ophthalmologic exam, including fundoscopy was normal. Inhibitory control was impaired. The phono audiological assessment revealed moderate dysarthria, with impairment of phonation and articulation, in addition to sporadic gagging. He only tolerates liquid food, showing dysphagia to solids. Laboratory tests, including full blood count, biochemical evaluation, renal and liver function tests were normal. MRI of P3 was evaluated by researchers but is not available in digital mode. A T2 weighted brain MRI demonstrated symmetrical central hyperintensity surrounded by hypointense signal in globus pallidus, consistent with the "eye-of-the-tiger" sign. T2 demonstrated low signal in corresponding areas from iron deposition. The molecular analysis of exon 5 of the PANK2 gene revealed the presence of the two heterozygous pathogenic variations: c.[965A > G] (p.Glu322G1y); c.[1070G > C] (p.Arg357Pro).

### Procedures and assessments

The three patients were evaluated both by a pediatric neurologist and geneticist. Biochemistry and hematologic tests were performed, and patients underwent 3 T MRI prior to the treatment.

**Fig. 2:**
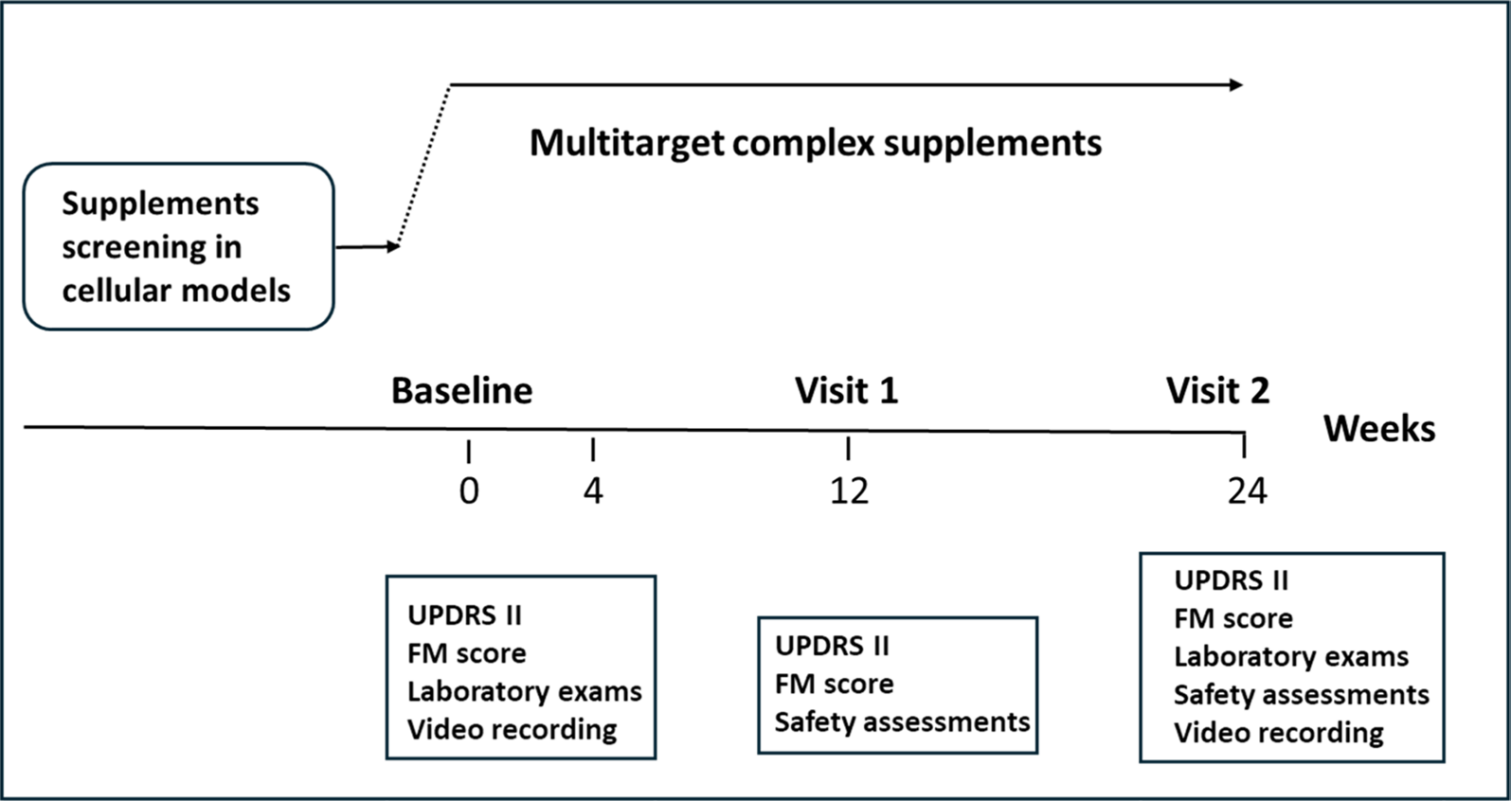
Flow chart of the study design. The treatment period lasted for 24 weeks

The following scales were applied before the treatment and 24 weeks later during the follow up period: Unified Huntington’s Disease Rating Scale, Unified Parkinson’Disease Rating Scale Part II (UPDRS II); Fahn-Marsden (FM) Scale (Fig. 2).

## Results

### Effect of multitarget complex supplements at cellular level

To support the clinical therapeutic approach, the effect of multitarget complex supplements (pantothenate, pantethine, Omega 3 and vitamin E) on iron accumulation, PANK2 and mtACP expression levels and lipid peroxidation in fibroblasts derived from patients P1, P2 and P3 were examined.

### Multitarget complex supplements reduced *iron* accumulation in fibroblasts derived from PKAN patients

**Fig. 3:**
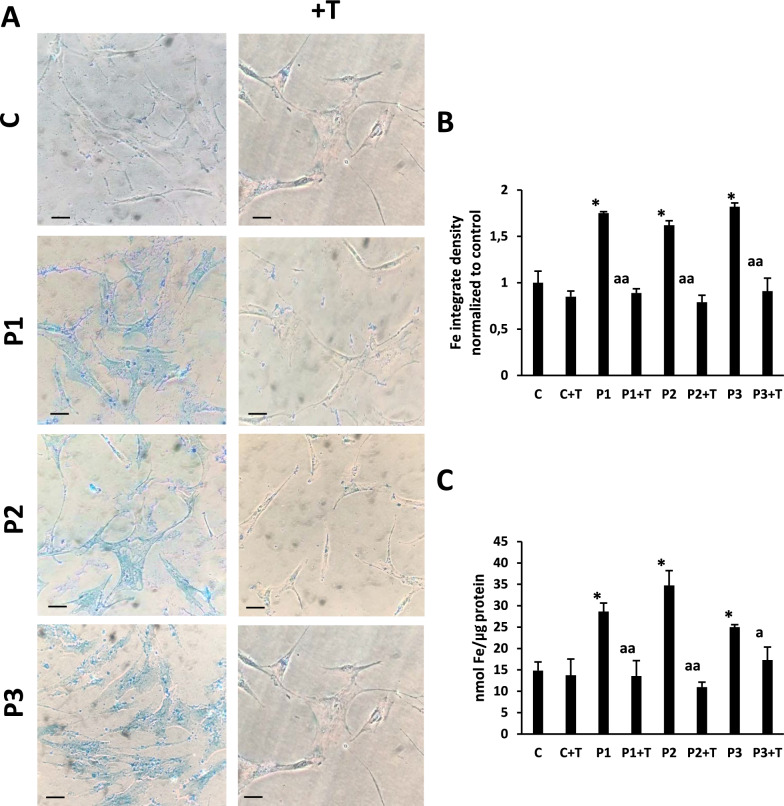
Effect pantothenate, pantethine, omega-3 and vitamin E supplementation on iron accumulation in three mutant *PANK2* cells. (**A**) Control (C1) and three PKAN fibroblast cell lines (P1, P2 and P3) were treated with 5 µM pantothenate, 5 µM pantethine, 5 µM vitamin E and 5 µM omega 3 for 10 days (+ T). Then, cells were stained with Prussian Blue as described in Material and Methods and examined by bright-field microscopy. Scale bar = 15 µm. (**B**) Quantification of Prussian Blue staining (**C**) Iron content determined by ICP-MS. Data represent the mean ± SD of three separate experiments. Significance between PKAN and control fibroblasts is represented as ^*^p < 0.01 fibroblasts and ^a^*p* < 0.05, ^aa^*p* < 0.01 between untreated and treated fibroblasts

As altered iron metabolism is one of the main characteristics of PKAN, we first examined intracellular iron accumulation and the effect of multitarget complex supplements (5 µM pantothenate, 5 µM pantethine, 5 µM vitamin E and 5 µM omega 3) by Prussian Blue staining in control and PKAN fibroblasts P1, P2 and P3. Iron staining was significantly increased in PKAN affected cells (Fig. 3A) which was significantly reduced in PKAN treated cells with multitarget complex supplements (Fig. 3A and B). Iron accumulation in patient fibroblasts and its elimination by supplements were confirmed by ICP-MS (Fig. 3C).

### Multitarget complex supplements increased PANK2 and mtACP expression levels.

**Fig. 4:**
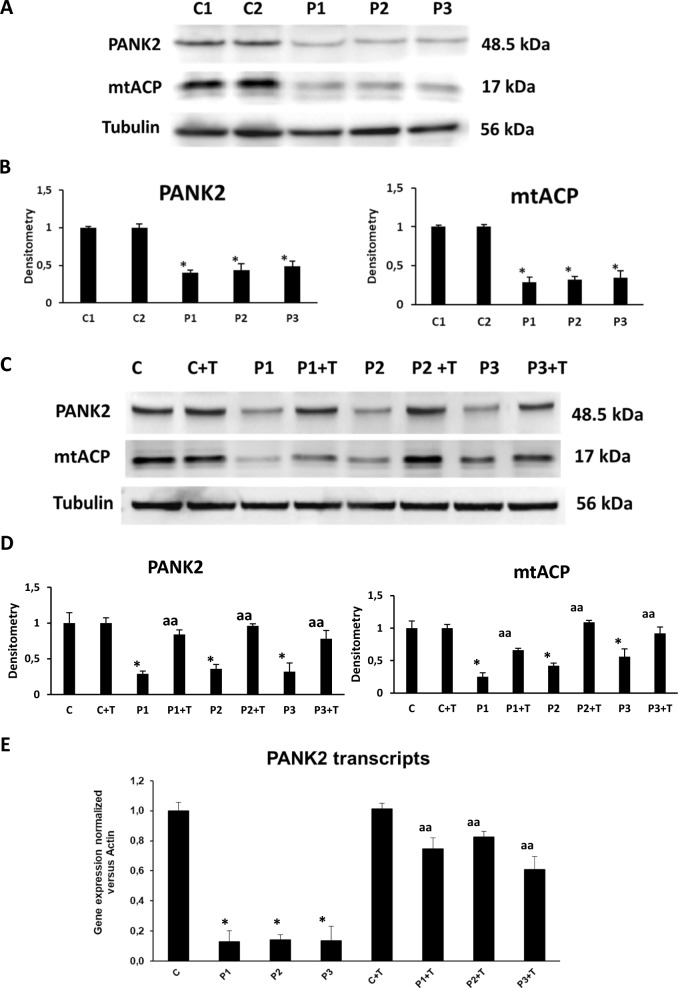
Effect of pantothenate, pantethine, omega-3 and vitamin E supplementation on PANK2 and mtACP expression levels. (**A**) Expression levels of PANK2 and mtACP in Control (C1 and C2) and PKAN cells (P1, P2 and P3) assessed by Western blotting. (**B**) Quantification of PANK2 and mtACP expression levels. (**C**) Controls (C1) and patient P1 fibroblasts were treated with 5 µM pantothenate, 5 µM pantethine, 5 µM vitamin E and 5 µM omega 3 for 10 days (+ T). PANK2 and mtACP protein expression levels were analysed by Western blotting. Tubulin was used as loading control. (**D**) Densitometry of the Western blotting of PANK2 and mtACP under pantothenate, pantethine, omega-3 and vitamin E supplementation. (**E**) *PANK2* transcripts were quantified by RT-qPCR. Data represent the mean ± SD of three separate experiments. *p < 0.01 between PKAN patients and controls. ^a^*p* < 0.05 and ^aa^*p* < 0.01 between untreated and treated fibroblasts. A.U., arbitrary units. Unedited and uncut blots are shown in Supplementary Figs. 1 and 2

Next, we analyzed PANK2 and mtACP expression levels in patient-derived fibroblasts. As shown by Western-blot analysis in Fig. 4A and B, PANK2 and mtACP expression levels were markedly reduced in PKAN fibroblasts while normal expression levels were present in control fibroblasts.

We next examined if the treatment with multitarget complex supplements (5 µM pantothenate, 5 µM pantethine, 5 µM vitamin E and 5 µM omega 3) was able to stabilize the mutant enzyme and increase mtACP expression levels in patient fibroblasts. The treatment 5 µM pantothenate, 5 µM pantethine, 5 µM vitamin E and 5 µM omega 3 increased markedly PANK2 and mtACP expression levels in P1, P2 and P3 fibroblasts (Fig. 4C and D). In addition, to assess if supplementation had a positive effect at the transcriptional level, we examined *PANK2* RNA expression levels by RT-qPCR. *PANK2* RNA levels were significantly reduced in P1, P2 and P3 mutant fibroblasts (Fig. 4C). Interestingly, treatment with 5 µM pantothenate, 5 µM pantethine, 5 µM vitamin E and 5 µM omega 3 markedly increased *PANK2* RNA levels in the three PKAN fibroblasts cell lines.

### Multitarget complex supplement reduced lipid peroxidation

**Fig. 5:**
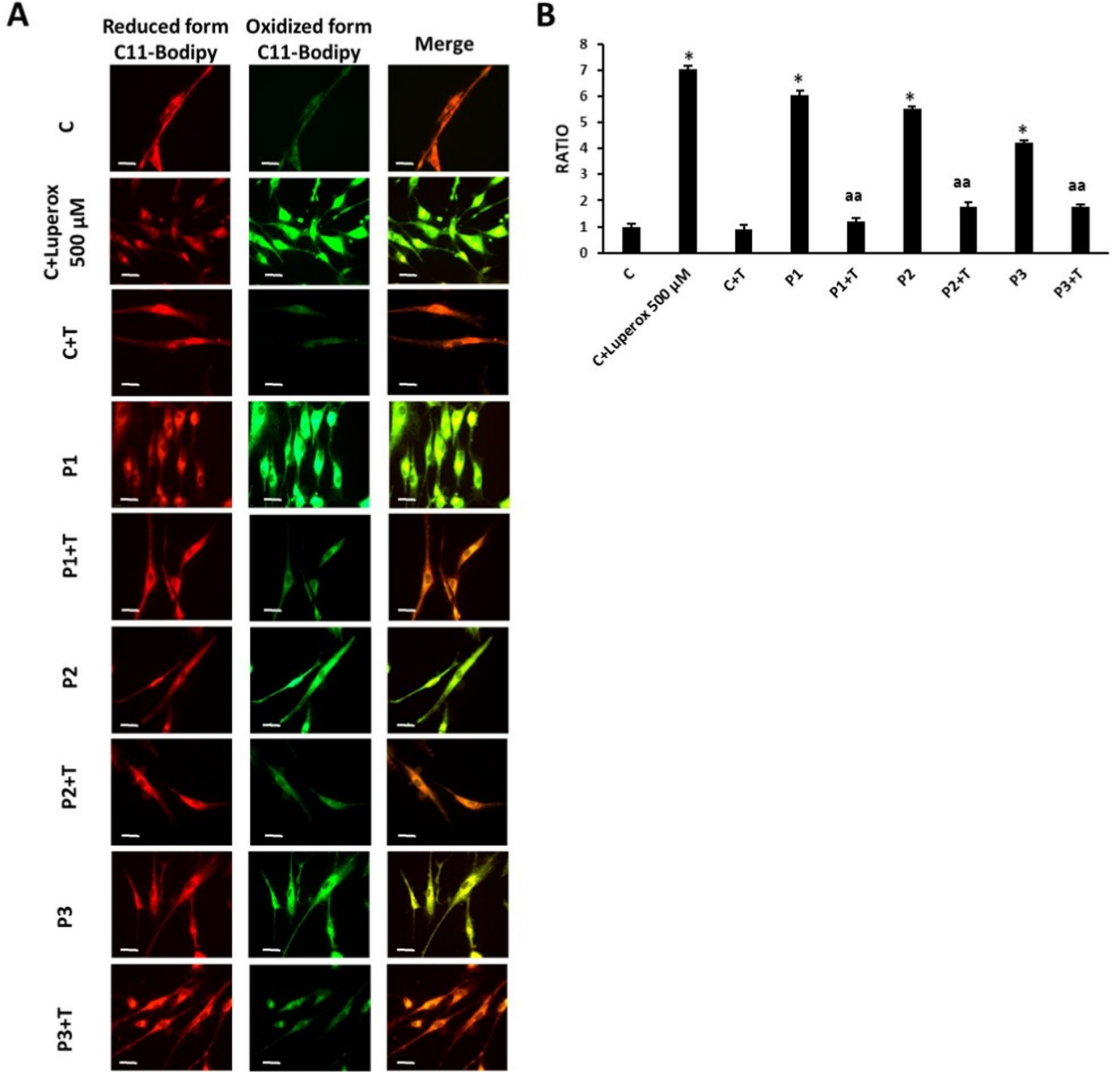
Effect of pantothenate, pantethine, omega-3 and vitamin E supplementation on lipid peroxidation. Control and PKAN fibroblasts (P1, P2 and P3) were treated with 5 µM pantothenate, 5 µM pantethine, 5 µM vitamin E and 5 µM omega 3 for 10 days (+ T). (**A**) Representative images of lipid peroxidation in treated and untreated control and PKAN cells using BODIPY® 581/591 C11 staining. Control cells treated with Luperox® (500 μM) for 15 min were used as a positive control of lipid peroxidation. Scale bar = 15 μm. (**B**) Ratio of the oxidized BODIPY-C11 signal (green) to reduced BODIPY-C11 signal (red). Data represent the mean ± SD of three separate experiments (50 cell images for each condition). *p < 0.01 between PKAN patients and controls. ^aa^*p* < 0.01, between untreated and treated fibroblasts. A.U., arbitrary units

The levels of lipid peroxidation in treated and untreated fibroblasts were assessed by BODIPY™ 581/591 C11, a lipid peroxidation sensor. PKAN fibroblasts P1, P2 and P3 showed increased lipid peroxidation levels compared to control cells and multitarget complex supplements (5 µM pantothenate, 5 µM pantethine, 5 µM vitamin E and 5 µM omega 3) were able to significantly reduce them in mutant fibroblasts (Fig. 5A and B).

### Effect of multitarget complex supplements on PDH activity in PKAN fibroblasts

**Fig. 6:**
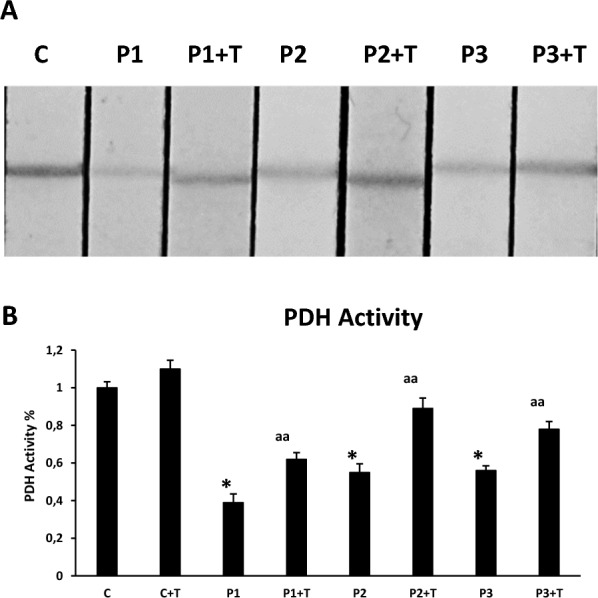
Effect of pantothenate, pantethine, omega-3 and vitamin E supplementation on PDH activity. Control and PKAN fibroblasts (P1, P2, P3) were treated with 5 µM pantothenate, 5 µM pantethine, 5 µM vitamin E and 5 µM omega 3 for 10 days (+ T). (**A**) PDH activity in whole cellular extracts was measured as described in Material and Methods. (**B**) Quantification of PDH activity. Data represent the mean ± SD of three separate experiments. *p < 0.01 between PKAN patients and controls; ^aa^*p* < 0.01 between untreated and treated cells

Next, we focused on the pathological alterations potentially induced by mtACP deficiency. Thus, as mtACP is essential for lipoic acid synthesis by mitochondrial FAS II [23], we explored protein lipoylation in control and PKAN fibroblasts. Lipoic acid is a cofactor central to cellular metabolism [24, 25]. As a lysine posttranslational modification on particular components of enzymatic complexes, this functional group is required for the activities of these multimeric complexes such as PDH [26, 27]. As is shown in Fig. 6A and B, PDH was drastically reduced in PKAN fibroblasts. Interestingly, multitarget complex supplements were able to restore partially PDH activity (Fig. 6A and B) in mutant PANK2 fibroblasts.

### Treatment and follow-up of patients

Patients were treated with baseline medications (Table 1) plus adjuvant supplements which included high doses of pantothanate, pantethine, Omega 3 fatty acid and vitamin E, for 6 months. The primary endpoints were change of the Unified Parkinson’s Disease Rating Scale (UPDRS) II score from baseline to week 24 after treatment.

**Table 1:** Clinical and genetic characteristics of the PKAN patients (P1, P2 and P3)

Patient ID	Sex	Age at onset (years)	Symptoms at base line	Score of UPDRS II at W0	Score of UPDRS II at W24	Baseline medication	Variants in *PANK2* (change of nucleotide)	Change of amino acid
P1	F	3	Walking difficulty	23	10	Trihexylphenidyl hydrochloride, Bacoflen, Gabapentin	c.[1168-3C > G] c.[1263dupG]	Splicing defect p.Gln422Alafs*21
P2	F	14	Distonic foot	36	30	Trihexylphenidyl hydrochloride, Bacoflen	c.[950G > C] c.[1231G > A]	p.Gly317Ala p.Gly411Arg
P3	M	20	Dystonia in lower limbs	33	33	Baclofen, Bideperiden Bromazepan	c.[965A > G] c.[1077G > C]	p.Glu322Gly p.Arg357Pro

Patients received all the supplements through oral administration. The dose of pantethine started at 600 mg per day, gradually increased to 1200 mg per day within 5 days and maintained at 1200 mg per day (divided into two dosage), pantothenate was started at 500 mg and gradually increased until 6 g per day in three dosages, within 4 weeks; Omega 3 started at 2000 mg in two dosage and vitamin E 400UI three times a week. During the study, the dosage of other drugs taken by patients at baseline remained unchanged. Patients were contacted weekly by phone to find out if there were any drug related adverse events in the first 5 weeks and were visited at week 12 (W12) and 24 (W24).

Assessments of efficacy were conducted using the following measures: (1) UPDRS I-III was used to assess motor symptoms, including mental behavior, daily activity, and motor sections by generating individual scores and a total score (0–124); (2) FM scale was used to measure the severity of dystonia in nine body regions (eyes, mouth, neck, trunk, swallowing, speech, and each upper and lower extremities) by generating individual scores and a total score (0–120); (3) Parental clinical impression scale, which is a subjective instrument consists of a single question, was used to ask parents to rate their children’s condition on a scale from 1 (markedly better) to 5 (markedly worse) compared with how they felt at the baseline; (4) Blinded video rating was independently performed by the pediatric neurologist and geneticist. Conflicts in opinions were resolved by consulting another blind neurologist to make a decision. Patient’s condition at W24 was rated on a scale from 1 (markedly better) to 5 (markedly worse) compared with how they felt at baseline through watching the videos (the actions included opening and closing eyes, opening and closing mouth, turning head, eye movements, tapping the index finger with the thumb, stretching and bending arms, placing both arms flexed at the elbow in front of the chest, stamping the foot, finger–nose test, turning body, walking, eating something, writing, and speaking).

Safety assessments were conducted at each visit through data collection of adverse events and clinical laboratory tests including liver function, renal function, and complete blood count at week 24. The primary endpoints were the change of UPDRS II and FM scores from baseline to W24 after treatment.

The baseline medications usually taken by the patients included trihexyphenidyl hydrochloride, clonazepam and baclofen. The supplementation with pantothanate, pantethine, Omega 3 fatty acid and vitamin E was well tolerated during the 24-week treatment. Liver and renal function, as well as blood routine remained normal during the study period. One patient (P1) showed mild nausea and diarrhea but with no need for dose reduction or therapeutic regimen modification. Another patient (P3) presented diarrhea in the first week requiring lowering the dosage of pantothenate for one more week (500 mg). Adverse events did not recur over the weeks of evaluation.

### Primary endpoints of efficacy

To clarify whether the treatment could slow the progression of motor dysfunction in PKAN patients, we compared the rate of increase in score of UPDRS II and FM 24 week before and after the treatment. The rates of increase in individual score of UPDRS II and FM scores were slowed after the treatment or at least remained the same (Table 2).

**Table 2:** FM score at baseline (W0) and W24 of PKAN patients (P1, P2 and P3)

ID	W0	W24	ΔW24-W0
P1	23.0	15.0	−8.0
P2	98.0	74.0	−24.0
P3	56.0	56.0	0.0

No statistical analysis was performed.

The results indicated that although treatment couldn’t significantly improve the motor function, it may delay the progression of motor dysfunction. The patients showed improvement in dystonic symptoms, although language, cognition, and functional independence showed no improvement.

## Discussion

PKAN is a progressive neurodegenerative disorder that affects movement, balance, speech, vision, cognition, and behavior, and it arises from pathological variants in the *PANK2* gene [19]. To date, treatment has been symptomatic and there are no approved therapies that target the underlying mechanistic defects in PKAN.

Precision medicine is the tailoring of medical treatment to the unique characteristics of each patient. In contrast to the traditional "one-drug-fits-all" approach, the use of precision medicine in the treatment of neurodegenerative disorders such as PKAN appears to be very promising [28–30]. In this context, the development of a precision medicine approach based on cellular models derived from PKAN patients could provide an excellent opportunity to identify optimal treatments.

Since the precise mechanism of PKAN has yet to be discovered, most current treatments aim to alleviate symptoms to some extent. Any treatment's ability to cross the blood–brain barrier determines its therapeutic efficacy. As a result, multitarget therapeutics using various compounds and formulations can aid in achieving the optimal concentration in target tissues such as the brain [28].

We observed that the combination of pantothenate, pantethine, Omega 3 fatty acids and vitamin E may have potential as an adjuvant disease-modifying intervention in PKAN patients with residual enzyme levels [18, 28]. Furthermore, the combined treatment is capable of increasing PANK2 and mtACP expression levels in patient-derived fibroblasts.

### Pantothenate

Pantothenate, also known as vitamin B5, is a precursor of the CoA biosynthesis pathway [31]. The molecule is widely distributed in living organisms [32] and pantothenate deficiency in humans may occur only as a consequence of severe malnutrition. In the cells, CoA synthesis begins with the phosphorylation of pantothenate to 4`-phosphopantothenate (P-Pan) by PANK. This first reaction represents the main rate-limiting and control step in the entire process of CoA biosynthesis [33]. High-dose pantothenate has been proposed as a potential compound for a subset of patients who have PANK2 enzyme deficiencies that retain some residual function [10, 14]. This strategy is based on the premise that a functionally weak enzyme may perform better at higher substrate concentrations.

Pantothenate effectiveness has been previously examined in two in vitro cell models, one involving primary skin fibroblasts derived from PKAN patients and the other involving induced neurons that were generated from patient fibroblasts by direct reprogramming [10]. Pantothenate supplementation resulted in increased CoA levels in mitochondria of fibroblasts with residual PANK2 enzyme expression but not in patient fibroblasts without residual PANK2 enzyme expression. In addition, pantothenate was capable of eliminating iron accumulation in mutant neurons generated from the patients’ fibroblasts with residual PANK2 expression. These in vitro studies are significant because they show that high-dose pantothenate may be beneficial for specific patients. However, this approach is applicable only to patients who have some residual PANK2 function [18]. In addition, it remains unclear whether pantothenate could be delivered in sufficient amounts to have the desired functional effect on the enzyme in the human brain in vivo.

### Pantethine

Pantethine is a dimeric form of pantetheine that was shown to rescue disease phenotypes in bacteria [34], Drosophila [35], zebrafish [36] and mouse PKAN models [37]. A recent clinical trial evaluated the safety and efficacy of 60 mg/day of pantethine in pediatric PKAN patients over a 24-week period [38]. Serum CoA levels were not altered before or after treatment and there was no significant change in the primary clinical endpoints studied. The limited efficacy of pantethine in affected patients may be due to poor pharmacokinetic properties. Pantethine is highly unstable in serum and rapidly converts to vitamin B5 and cysteamine instead of CoA [39, 40]. More research may be necessary to determine the effectiveness of this therapeutic strategy. However, because pantethine is also a source of pantothenate, combining pantethine and pantothenate can increase the concentration of the substrate more than a single treatment.

### Vitamin E

The oxidative status has been previously analyzed in PKAN fibroblasts [41]. Signs of oxidative stress were detected in cells from patients, and ROS production was increased in these cells after exposure to iron. In agreement with these findings, our group found increased amount of carbonylated proteins and mitochondrial lipid peroxidation in PANK2 mutant fibroblast which were prevented by the treatment with pantothenate in responsive mutant cells with residual PANK2 levels [10].

Lipid peroxidation can be described as a process under which oxidants such as free radicals or nonradical species attack lipids containing carbon–carbon double bond(s), especially polyunsaturated fatty acids (PUFAs) that involve hydrogen abstraction from a carbon, with oxygen insertion resulting in lipid peroxyl radicals and hydroperoxides [42].

The process of lipid peroxidation consists of three steps: initiation, propagation, and termination [43]. In the lipid peroxidation initiation step, prooxidants such as the hydroxyl radical abstract allylic hydrogen, resulting in the formation of the carbon-centered lipid radical. During the propagation phase, a lipid radical (L•) quickly reacts with oxygen to form a lipid peroxy radical (LOO•), which extracts a hydrogen from another lipid molecule, resulting in a new L• (which continues the chain reaction) and lipid hydroperoxide (LOOH). In the termination reaction, antioxidants such as vitamin E donate a hydrogen atom to the LOO• species, forming a vitamin E radical that reacts with another LOO• to produce nonradical products. Once lipid peroxidation begins, a chain reaction will occur, resulting in termination products. The chemistry involved in each of these steps is covered in detail elsewhere [44].

Lipid peroxidation generates multiple reactive aldehydes, such as malondialdehyde (MDA) and 4-hydroxynonenal (4-HNE) [42]. Proteins and DNA are among the substrates most vulnerable to aldehyde modification. MDA and 4-HNE adducts play important roles in a variety of cellular processes and can participate in secondary deleterious reactions by promoting intramolecular or intermolecular protein/DNA crosslinking, which can cause profound changes in the biochemical properties of biomolecules and facilitate the development or worsening of various pathologies. Lipid peroxidation (LPO) is linked to the development of many neurodegenerative diseases (NDDs), including Alzheimer's disease (AD), Parkinson's disease (PD), and amyotrophic lateral sclerosis (ALS), all of which have elevated levels of LPO products and LPO-modified proteins [45].

Vitamin E is a well-known chain-breaking antioxidant with the specific function of preventing lipid peroxidation in membrane systems [46]. In addition, vitamin E is necessary for neurological function. This fact, combined with a growing body of evidence indicating that neurodegenerative processes are associated with oxidative stress, leads to the convincing idea that the antioxidant properties of vitamin E may prevent and/or cure several neurological disorders [47].

Studies on human and animal models of vitamin E deficiency established the critical roles of the vitamin in protecting the central nervous system, particularly the cerebellum, from oxidative damage and motor coordination deficits [48]. Vitamin E's function has traditionally been attributed to its antioxidant properties. This notion is based on the vast literature that documents tocopherol's efficacy in neutralizing unstable lipid peroxy-radicals generated by polyunsaturated fatty acids [49].

The PKAN pathogenesis is directly related to the overproduction of ROS and unbalanced mitochondrial redox, which may trigger a neuronal death cascade [50]. Lipid peroxidation and oxidative status changes (increased ROS production), mitochondrial impairment (including defects in mitochondrial respiration and electrophysiological properties), and premature cell death have all been observed in fibroblast and neuronal cells derived from PKAN patients [11, 41]. Thus, inhibiting neuronal oxidation may slow the progression and severity of PKAN; antioxidant vitamins, such as vitamin E, appear to be promising adjuvant treatments.

### Omega-3 fatty acids

Omega-3 fatty acids are polyunsaturated fatty acids (PUFs) that are commonly found in fish, vegetable oils, nuts, flax seeds, and leafy vegetables. They are an essential component of cell membranes, ensuring stability, fluidity, synaptic connectivity, and avoiding the oxidative stress caused by ROS [51].

The beneficial effects of omega-3 fatty acids have been demonstrated by numerous studies due to their involvement in various biochemical functions, such as gene expression, intracellular signaling, cell membrane fluidity, and the synthesis of anti-inflammatory mediators [52–54]. In addition, Omega-3 has been shown to reduce lipid peroxidation [55, 56]

There is an accumulation of scientific evidence on the possible efficacy of PUFA supplementation in neurodegenerative disorders. [57, 58], such as Parkinson’s (PD) and Alzheimer's disease (AD) [59].

While there is no known cure for neurodegenerative diseases, dietary recommendations may help manage symptoms and halt the progression of cognitive and physical impairment.

In this regard, combination drugs that act on multiple targets simultaneously can be better at controlling complex disease systems, less susceptible to drug resistance, and are the standard treatment in many therapeutic fields [60, 61]. Many monotherapies have limitations that can be overcome by attacking the disease system in multiple ways [62]. Thus, multitarget therapy is more effective and less susceptible to adaptive resistance because biological systems cannot compensate for the effects of multiple drugs simultaneously.

Combination searches using active pharmaceutical ingredients can be especially useful, as potential synergies identified by these screens can rapidly advance in preclinical and clinical development. [63]. Furthermore, the combination of substances with identified biological molecular targets may disclose unanticipated interactions between disease-related pathways [64]. Ultimately, a deeper comprehension of the biology of diseases may result from this route-oriented approach to target discovery [65].

Neurodegenerative diseases, such as PKAN, are a group of progressive disorders characterised by structural and functional degeneration of the human nervous system. Impaired mitochondrial function, excessive oxidative stress in the human brain, genetic factors, and dysfunction in human brain metabolism contribute to the progression of neurodegenerative diseases [66]. Regarding this, multitarget therapy promises to address the multifaceted complexity of neurodegenerative diseases [67, 68]. In recent years, the use of multitarget directed ligands has emerged as a powerful strategy in the development of potential therapies for neurological disorders [69]. A major advantage of multitarget therapies is their ability to act on multiple targets involved in the progression of these diseases compared to a single-target concept.

Despite developments in understanding the genetics, pathophysiology, and clinical presentation of PKAN in the last years there is no promising disease-modifying therapies for PKAN yet [28, 70]. Therapies usually used in patients included Deep Brain Stimulation (DBS), deferiprone, fosmetpantotenate (RE-024), 4′-phosphopantetheine (CoA-Z) [71].

In this pilot study of 24 weeks of treatment with adjuvant supplements, we found an improvement in motor function in two of the three PKAN patients. However, we did not find any improvement in one of the 3 patients, although there was also no worsening, suggesting that the intervention could delay the progression of motor dysfunction. This may indicate that patients with relatively mild motor handicap at baseline were more likely to improve after the treatment. Another factor that may contribute to supplements effectiveness is the onset of the disease. Thus, the two patients who improved, were of an early and an intermediate onset type, whereas the "stable" patient showed late onset. Therefore, the time of onset might also play a role.

It is well known that the time of onset of PKAN significantly influences the disease progression [1]. PKAN is generally categorized into two forms based on the age of onset: classic (early onset) and atypical (late-onset). Classic PKAN, with an onset typically before the age of 10 years (P1), is characterized by rapid disease progression. Patients with early onset PKAN often experience severe neurological impairment, including dystonia, dysarthria, and loss of independent ambulation, within a few years of symptom onset. The median interval between disease onset and the occurrence of oromandibular dystonia, generalized dystonia, and loss of independent ambulation is notably shorter in early-onset patients compared to late-onset patients. Additionally, early-onset PKAN is associated with a higher mortality rate, with about 20% of patients dying at a median age of 12.5 years, approximately 9.5 years after disease onset [1]. It is important to note that even with early onset, P1 presented stabilization of the course of the disease, with no periods of worsening over the years (besides the study period).

In contrast, atypical PKAN, with an onset typically after the age of 10 years, progresses more slowly. Patients with late-onset PKAN generally exhibit mild symptoms and a prolonged disease course. The median interval between disease onset and significant milestones such as oromandibular dystonia, generalized dystonia, and loss of independent ambulation is longer in late-onset patients.The mortality rate in late-onset PKAN is significantly lower, with only about 2% of patients dying during the follow-up period [1]. P2 presented symptoms since the age of 14 years, and although the disease course was more prolonged, she lost independent ambulation since the age of 18 years. After starting the multi-target complex, she recovered some important functions such as speech (slightly involved, easily understood) and less generalized dystonia (Supplementary Fig. 3), allowing her to use the toilet in a sitting position.

Patient P3 had a later onset but with similar symptoms to P2, characterized by bradykinesia, rest tremor, and rigidity, which are common features of parkinsonism observed in PKAN patients. Additionally, dystonia was frequently present in the three subjects and was the predominant symptom, often occurring alongside parkinsonism. P3 didn’t have improvement or worsening.

In summary, adjuvant supplements treatment in PKAN patients proved to be safe and well-tolerated with only transient diarrhea, which resolved after dosage adjustment and has not recurred with gradually increasing doses. Safety and tolerability, however, remain to be confirmed in larger numbers of patients. Some limitations of this work are that it was a single-arm, open-label study and the number of patients was limited. In addition, the treatment duration was not long.

## Conclusion

In this manuscript we provide evidence that the complementation of the baseline neurological medication of PKAN patients with pantothenate, pantethine (aiming to increase PANK2 and mtACP levels) and omega 3 and vitamin E (targeting oxidative stress and lipid peroxidation) could help in improving or delaying the progression of motor dysfunction. Novel targeted treatments are extremely needed to retard or stop disease progression and to optimize the quality of life in PKAN. Larger patient cohorts should be studied to determine the therapeutic effects of this adjuvant multitarget complex supplements in PKAN.

## Supplementary Information

Additional file1

## Data Availability

Data supporting the findings of this study are not openly available due to reasons of sensitivity and to protect the privacy of individuals; however, data are available from the corresponding author upon reasonable request. Data are located in controlled access data storage at Pablo de Olavide University (https://jazmin.upo.es/bscw/bscw.cgi).

## Abbreviations

AASDHPPT: L-aminoadipate-semialdehyde dehydrogenase-phosphopantetheinyl transferase.
AASS: Alpha-aminoadipic semialdehyde synthase
mtACP: Mitochondrial acyl carrier protein
ALDH1L2: Mitochondrial 10-Formyltetrahydrofolate Dehydrogenase
CoA: Coenzyme A
DAPI: 4 ',6-Diamidino-2-fenilindol
FSE: Fast spin eco
NBIA: Neurodegeneration with brain iron accumulation
PANK2: Pantothenate kinase 2
PDH: Pyruvate dehydrogenase
PKAN: Pantothenate kinase-associated neurodegeneration
